# Post-Marketing Safety Surveillance of Influenza Vaccines in Anhui Province, China, 2016–2025

**DOI:** 10.3390/vaccines14060548

**Published:** 2026-06-21

**Authors:** Fanya Meng, Sicheng Wei, Binbing Wang, Xianwei Luo, Jiabing Wu

**Affiliations:** 1 Anhui Provincial Center for Disease Control and Prevention, Hefei 230601, China; mfy@ahcdc.com.cn (F.M.); wbb@ahcdc.com.cn (B.W.); lxw@ahcdc.com.cn (X.L.); 2 School of Public Health, Wannan Medical University, Wuhu 241002, China; 20259110@stu.wnmc.edu.cn

**Keywords:** China, influenza vaccination, adverse events following immunization, vaccine safety, surveillance

## Abstract

**Background**: China’s influenza vaccine (InfV) has undergone multiple iterations and numerous technological breakthroughs, providing tremendous impetus and solid support for the development of China’s health sector. As the number of vaccinated individuals continues to rise, the importance of ongoing surveillance and evaluation of vaccine safety has become increasingly prominent, forming part of efforts to maintain public trust in the national immunization program and ensure its sustainability. **Methods**: From 2016 to 2025, data on suspected adverse events following immunization (AEFIs) related to InfV administration were extracted from the Chinese National Immunization Information System (CNIIS). Data on InfV vaccination doses were obtained from the Anhui Provincial Immunization Information Management System. A descriptive statistical method was used to analyze the distribution characteristics of AEFIs, and the chi-square test was applied to evaluate differences in reporting rates. **Results**: Between 2016 and 2025, a total of 4026 AEFI reports related to InfV were monitored through the CNIIS. The overall reporting rate was 34.40 per 100,000 doses. Specifically, common adverse reactions and rare adverse reactions accounted for 95.88% (3860 cases) and 3.38% (136 cases), with reporting rates of 32.98 per 100,000 doses and 1.16 per 100,000 doses, respectively. Among common adverse reactions, the reporting rates of fever (axillary temperature ≥ 38.6 °C), local redness and swelling at the injection site (diameter > 5.0 cm), and local induration (diameter > 5.0 cm) were 9.62 per 100,000 doses, 1.96 per 100,000 doses, and 1.20 per 100,000 doses, respectively. Among rare adverse reactions, the reporting rates of allergic rash, angioedema, anaphylactic shock, febrile convulsions, anaphylactoid purpura, thrombocytopenic purpura, epilepsy, Guillain–Barré syndrome, and aseptic abscess were 0.98, 0.05, 0.03, 0.03, 0.02, 0.02, 0.01, 0.01, and 0.01 per 100,000 doses, respectively. No cases were reported for subunit inactivated influenza vaccine (IIV, Subunit). Statistically significant differences were observed in the reporting rates of allergic rash across different types of InfV (χ^2^ = 36.83, *p* < 0.05), with trivalent inactivated influenza vaccine (IIV3, Split) and trivalent live attenuated influenza virus vaccine (LAIV3) showing the highest reporting rates. Most adverse events following vaccination occurred within 24 h after inoculation. **Conclusions**: From 2016 to 2025, the overall reporting rate of AEFIs after InfV administration in Anhui Province was within an acceptable range. Common adverse reactions were common, while rare adverse reactions were few, mainly consisting of allergic reactions. These results indicate that InfV has a favorable safety profile, and continuous strengthening of AEFI surveillance for InfV and improvement of surveillance quality are warranted.

## 1. Introduction

The research and development of influenza vaccine (InfV) in China began with initial exploration in the 1950s and has undergone rapid iteration to date, achieving numerous technological leaps and breakthroughs in new formulations. Having obtained pre-qualification from the World Health Organization (WHO), Chinese InfV has entered the international market, ending dependence on imported vaccines and establishing a vaccine supply system adapted to the immune characteristics of the Chinese population. This provides core support for the implementation of the national influenza immunization program and is critical for reducing influenza incidence and easing the pressure of public health prevention and control. Influenza is a severe acute respiratory infectious disease harmful to human health, caused by influenza viruses and transmitted mainly through respiratory droplets and indirect contact. Its main clinical manifestations include fever, cough, headache, myalgia, sore throat, runny nose, and other symptoms [1]. Influenza outbreaks occur frequently in winter and spring. Due to the rapid mutation of the virus and the short duration of protective antibodies produced by the human body, people of all ages are generally susceptible to influenza viruses. Influenza viruses tend to cause clustered infections, and activities involving high-density human contact greatly increase the chance and speed of influenza virus transmission among populations. According to estimates by the WHO, annual seasonal influenza epidemics result in illness in 5–10% of adults and 20–30% of children globally, leading to 3–5 million severe cases and 290,000–650,000 respiratory disease-related deaths [2,3,4]. Influenza epidemics severely affect students’ academic performance and quality of life, and may also exert negative impacts on their mental health [5]. Influenza vaccination is an effective measure to prevent influenza and reduce the risk of influenza infection and severe complications among vaccinees [6]. The WHO recommends that all countries consider implementing seasonal influenza vaccine immunization programs to strengthen the prevention and control of influenza pandemics [7]. Currently, InfV is not included in China’s National Immunization Program (NIP), and related costs are mainly borne by vaccinees themselves. InfV is a nationwide non-expanded immunization vaccine in China, and voluntary self-paid influenza vaccination is carried out in all provinces across the country, not limited to Anhui. As public attention to influenza prevention in China continues to grow, the administration of InfV has become increasingly widespread, making the monitoring of AEFI critically important. Although pre-marketing clinical trials have confirmed the safety of the vaccine, continuous monitoring and in-depth analysis of post-marketing AEFI surveillance data are still required.

This study aimed to analyze post-marketing AEFI surveillance data of influenza vaccines (InfV) in Anhui Province from 2016 to 2025. By collecting and evaluating adverse reaction cases after vaccination, we compared the differences in AEFI profiles and reporting rates among different types of InfV to ensure the safety of influenza vaccines. This study adopted standardized research methods consistent with those used in Post-Marketing Safety Surveillance of HPV Vaccines in Anhui Province, China, 2017–2024.

## 2. Materials and Methods

### 2.1. Data Collection

Data on InfV-related AEFIs and the corresponding number of InfV doses administered in Anhui Province from 2016 to 2025 were obtained separately through the China National AEFI Surveillance System (CNAEFIS) and the Anhui Provincial Immunization Program Information Management System (Anhui IIMS).CNAEFIS was established in 2005. Affiliated with the Chinese Center for Disease Control and Prevention (China CDC) and headquartered in Beijing, China, it is a nationwide passive post-marketing vaccine safety surveillance system. Anhui IIMS was established in 2008, is affiliated with the Anhui Provincial Center for Disease Control and Prevention, and is based in Anhui, China. Descriptive analysis and chi-square test were used to monitor the distribution characteristics and reporting incidence of InfV-related AEFIs.

### 2.2. Vaccine Types and Vaccination Schedule

InfV approved for marketing in China include trivalent inactivated influenza vaccine (IIV3), trivalent live attenuated influenza virus vaccine (LAIV3), and quadrivalent inactivated influenza vaccine (IIV4). Both IIV3 and IIV4 comprise split vaccines and subunit vaccines. Trivalent influenza vaccine (subunit) [IIV3(Subunit)] and quadrivalent influenza vaccine (subunit) [IIV4(Subunit)] are combined as “influenza vaccine (subunit) [IIV(Subunit)]”. The 0.25 mL formulation of split IIV3 and IIV4 is indicated for individuals aged 6 months to 3 years. The 0.5 mL formulation, depending on the manufacturer, is indicated for people aged ≥ 6 months or ≥3 years, respectively. The 0.5 mL formulation of the IIV4 subunit vaccine is suitable for individuals aged ≥ 6 months. LAIV3 is administered at 0.2 mL per dose to people aged 3–17 years [8].

### 2.3. Definition and Classification of AEFIs

Adverse Event Following Immunization (AEFI) refers to any suspected adverse reaction or event occurring after vaccine administration that may be associated with vaccination. In accordance with national AEFI surveillance under the National Immunization Program [9], AEFIs are classified into adverse reactions, vaccine quality incidents, vaccination incidents, coincidental events, and psychogenic reactions. Adverse reactions are further subdivided into common adverse reactions and rare adverse reactions. Common adverse reactions are induced by the inherent characteristics of the vaccine and only cause transient physiological dysfunction in recipients following immunization. They mainly present as fever and local redness and swelling, and may be accompanied by systemic symptoms such as general discomfort, lassitude, loss of appetite, and fatigue. Rare adverse reactions refer to adverse drug reactions that result in damage to the tissues, organs or functions of recipients caused by qualified vaccines during or after standard vaccination, with no fault of any relevant party. Vaccine quality incidents are defined as damage to recipients’ tissues, organs or functions caused by vaccines of substandard quality after administration. Vaccination incidents refer to damage to recipients’ tissues, organs or functions resulting from violations of immunization protocols, immunization schedules, vaccine use guidelines or vaccination schemes during the immunization process. Coincidental events occur when vaccination is administered during the prodromal or latent stage of a disease, which then manifests shortly after inoculation. Such diseases are usually caused by infections or other factors rather than vaccine-related properties. Psychogenic reactions specifically refer to adverse reactions caused by psychological or mental factors after vaccination, which are unrelated to vaccine components [10,11].

### 2.4. Surveillance Methods

In this study, safety surveillance of InfV was mainly based on a passive surveillance system, namely the AEFI Surveillance Module of the China National Disease Prevention and Control Information System (CNAEFIS). Established in 2005, this system is a national post-marketing vaccine safety surveillance network [12]. It uses AEFI reports to detect known reactions, identify unknown reactions, and analyze potential risk factors. Responsible reporting entities include medical institutions, vaccination clinics, centers for disease control and prevention, adverse drug reaction monitoring centers, vaccine manufacturers and distributors, as well as relevant personnel. The system adopts a territorial management model. When a suspected AEFI occurs, it must be reported immediately to the county-level health and drug regulatory authorities. Most reports should be submitted via the AEFI Case Report Form within 48 h. Cases involving death, severe disability, clustering events, or reactions of public concern must be reported by telephone or other urgent means within 2 h. After verification, reports are submitted through the national immunization information system. Relevant agencies at all levels conduct real-time monitoring of the data. For fatal or mass AEFIs, reporting procedures in accordance with national regulations on public health emergencies must be followed.

### 2.5. Statistical Methods

Data sorting was completed by office spreadsheet software. All formal statistical hypothesis testing, rate calculation, and trend analysis efforts were performed using SPSS (version 25.0, IBM Corporation, Armonk, NY, USA), with α = 0.05 as the test level. A descriptive analysis was used to examine the distribution characteristics and reporting incidence of InfV-related AEFIs, and the chi-square test was applied to compare the reporting incidence of AEFIs. The formula for calculating the reported incidence of AEFIs is as follows: Number of reported AEFI cases following InfV vaccination/Number of administered InfV doses × 100,000 doses.

## 3. Results

### 3.1. InfV Vaccination

**Figure 1:**
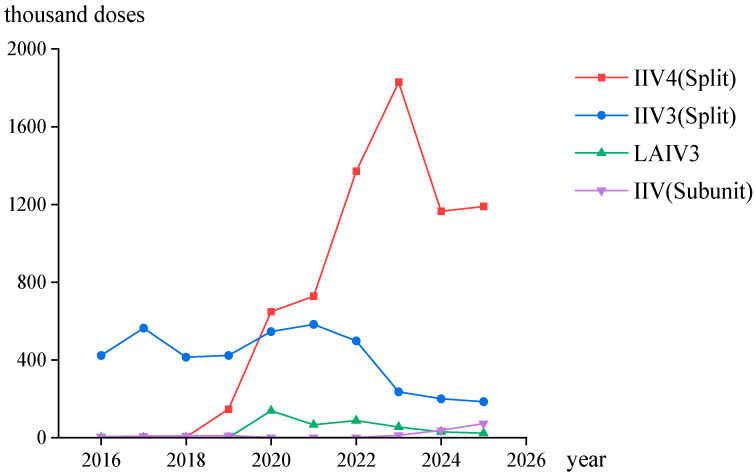
Number of doses administered for various types of InfV in Anhui Province, 2016–2025.

This study calculated the InfV vaccination rate in Anhui Province from 2016 to 2025 using the estimated data on permanent residents from the annual China Anhui Provincial Population Census Yearbooks [13] for each year as the denominator. The InfV vaccination coverage in Anhui Province was below 1% from 2016 to 2019. The vaccination rates in 2020, 2021, 2022, 2023, 2024 and 2025 were 2.19%, 2.25%, 3.19%, 3.48%, 2.34% and 2.42%, respectively. The InfV vaccination coverage in Anhui Province fluctuated before 2019, but increased significantly after 2019, reaching a peak of 3.48% in 2023. A total of 11,703,980 doses of InfV were administered across 16 cities in Anhui Province from 1 January 2016 to 31 December 2025. IIV4(Split) has become the most widely administered type of InfV at present, with a marked increase since 2019 and the highest vaccination volume in 2023; after peaking, it declined slightly in the following two years but remained at a high level.IIV3(Split) was the dominant vaccine type before 2020, and its vaccination volume became relatively low after being surpassed by IIV4(Split). LAIV3 has grown substantially since 2020. IIV(Subunit) showed a weak upward trend over the decade but maintained a low vaccination quantity, and surpassed LAIV3 in 2024 (Figure 1).

### 3.2. Classification of AEFIs

Between 2016 and 2025, a total of approximately 11,703,980 doses of InfV were administered in Anhui Province, China, during which 4026 cases of InfV-related AEFIs were reported, with an overall reported incidence of 34.40 per 100,000 doses. By etiological classification, the reported incidence was 32.98 per 100,000 doses (3860 cases) for common adverse reactions, 1.16 per 100,000 doses (136 cases) for rare adverse reactions, 0.21 per 100,000 doses (25 cases) for coincidental events, and 0.04 per 100,000 doses (5 cases) for psychogenic reactions. No vaccination accidents or vaccine quality issues are associated with adverse events were reported (Table 1). Among different types of InfV, statistically significant differences were observed in the reported incidence of both common and rare adverse reactions (χ^2^ = 76.81 and 41.71, respectively; both *p* < 0.05). The highest reported incidence of common adverse reactions was found for IIV4(Split), IIV3(Split), and IIV(Subunit), at 36.19, 29.28, and 28.09 per 100,000 doses, respectively. The highest reported incidence of rare adverse reactions was found for IIV3(Split), LAIV3, and IIV4(Split), at 2.04, 1.51, and 0.66 per 100,000 doses, respectively. No statistically significant differences were detected in the reported incidence of coincidental events and psychogenic reactions (χ^2^ = 6.27 and 3.94, respectively; both *p* > 0.05).

### 3.3. Distribution Characteristics of AEFIs

A total of 4026 AEFI cases associated with InfV were reported in Anhui Province from 2016 to 2025. The gender distribution of reported AEFIs showed no obvious difference, with males making up 49.74% and females 50.26%. When grouped by age, the differences in the reported incidence rates of AEFIs among different types of InfV across age groups were statistically significant: AEFI for IIV4(Split) occurred predominantly in the 3–17 years age group (χ^2^ = 4278.36, *p* < 0.05); for IIV3 (Split), AEFI occurred predominantly in the age group under 3 years (χ^2^ = 159.76, *p* < 0.05); for LAIV3, AEFI occurred predominantly in the 3–17 years age group (χ^2^ = 129.39, *p* < 0.05); and for IIV(Subunit), AEFI occurred predominantly in the 3–17 years age group (χ^2^ = 100.98, *p* < 0.05). When grouped by vaccination dose, adverse reactions following InfV immunization mostly appeared after the first dose, with 2939 cases, or 73.00% of all AEFI reports. Regarding the time to symptom onset after vaccination, the vast majority of AEFIs occurred within 24 h of injection, totaling 3804 cases, which accounted for 94.49% of the total (Table 2).

### 3.4. Clinical Diagnosis of Common Adverse Reactions

From 2016 to 2025, Anhui Province reported a total of 3860 cases of common adverse reactions, with a reporting incidence of 32.98 per 100,000 doses. These included 1127 cases of fever (axillary temperature ≥ 38.6 °C), 229 cases of local redness and swelling (diameter > 5.0 cm), and 140 cases of injection-site induration (diameter > 5.0 cm), with respective reporting incidences of 9.62, 1.96, and 1.20 per 100,000 doses. Within common adverse reactions, differences in the incidence of fever, local redness and swelling, and induration across different InfV types reached statistical significance (χ^2^ = 21.50, 172.98, and 145.43, respectively; *p* < 0.05). Among them, IIV4(Split), IIV3(Split), and IIV(Subunit) ranked high in the reporting incidence rates of fever, redness and swelling, and induration. The reporting incidence rates of fever were 8.74, 11.64, and 8.30 per 100,000 doses; those of redness and swelling were 2.60, 0.98, and 3.19 per 100,000 doses; and those of induration were 1.62, 0.52, and 2.55 per 100,000 doses, respectively (Table 3).

### 3.5. Clinical Diagnosis of Rare Adverse Reactions

A total of 136 rare adverse reactions following immunization were reported in Anhui Province from 2016 to 2025, with a reporting incidence of 1.16 per 100,000 doses. Allergic reactions consisted of 115 cases of allergic rash, 7 cases of angioedema, 4 cases of anaphylactic shock, 3 cases of febrile convulsions, 2 cases of anaphylactoid purpura, and 2 cases of thrombocytopenic purpura. Additionally, one case each was reported for epilepsy, Guillain–Barré syndrome, and aseptic abscess. No adverse events were reported for IIV(Subunit). The incidence of allergic rash differed significantly across various InfV types (χ^2^ = 36.83, *p* < 0.05), with the highest reporting rates seen in IIV3(Split) and LAIV3. For angioedema, anaphylactic shock, febrile convulsions, and other reactions, no significant between-group differences were detected (χ^2^ = 5.82, 1.94, 6.78, respectively; *p* > 0.05) (Table 4).

## 4. Discussion

Influenza poses a major public health threat to global human health, which is closely associated with the high variability, rapid transmission, and widespread susceptibility of influenza viruses among the general population. Influenza vaccination is one of the key measures to prevent severe illness, hospitalization, and death caused by influenza. With the continuous improvement of influenza vaccination services in China and the gradual expansion of vaccination coverage among priority populations, the importance of immunization in influenza prevention and control has become increasingly prominent. According to a study on vaccine effectiveness (VE) of influenza vaccine among inpatients aged ≥60 years with respiratory diseases in Beijing from 2013 to 2019, elderly individuals aged ≥60 years who received influenza vaccination had a lower risk of influenza-related death than those who were unvaccinated [14]. Among children aged 6 months to 5 years, vaccination could reduce the risk of influenza-related severe hospitalization by approximately 73%, fully demonstrating the protective value of timely vaccination among priority populations [15]. In addition, a global modeling study published in The Lancet predicted that if influenza vaccination coverage among global priority populations could reach 70–100%, combined with respiratory protection, early antiviral treatment, and surveillance and early warning, hundreds of thousands of influenza-related deaths could be avoided annually [2].

Since China standardized influenza vaccination, the vaccination rate among the general population has increased year by year. In some regions, free InfV is provided for key populations such as those aged 60 and above [16]. However, the overall vaccination rate in China remains low, at only approximately 3%, which is significantly lower than the average level of developed countries [17]. A cross-sectional survey conducted in the United States in May 2020 showed that the influenza vaccination rate reached as high as 53% [18]. The influenza vaccination rates in developed countries such as those in Europe and America, as well as some developing countries, even stood at 60–70%, and the rate among the elderly and medical workers exceeded 90% [19]. This indicates that there is still substantial room for improvement in China’s influenza vaccination rate. The influenza vaccination rate in Anhui Province has risen significantly since 2020, increasing from less than 1% to 3.48%. These data demonstrate that Anhui Province has made remarkable progress in boosting influenza vaccination coverage, yet it still lags behind that of some eastern provinces and falls far short of the threshold required to achieve herd immunity against influenza. Sustained efforts are still needed to attain higher vaccination targets in the future.

The reduction in the number of influenza vaccine doses administered across different vaccine types from 2023 to 2025 may be attributed to the following reasons: After large-scale population respiratory virus prevention during COVID-19, public overall attention to routine influenza vaccination declined year by year, leading to reduced active vaccination willingness. The influenza epidemic intensity in Anhui Province was relatively mild from 2023 to 2025, with fewer severe influenza cases reported in sentinel hospitals, weakening residents’ perceived risk of influenza infection. Short-term partial supply shortages of quadrivalent influenza vaccines occurred in some rural counties in 2024, temporarily restricting vaccination volume. Occasional online negative rumors about vaccine adverse reactions reduced parents’ vaccination intention for children and elderly guardians’ willingness to receive shots. Based on a survey of 31,786 users of a social media platform in China from 2021 to 2024, 55.29% of respondents reported vaccine hesitancy toward influenza vaccines. The reasons include concerns about common adverse reactions following vaccination, barriers to acquiring knowledge and information about influenza and vaccines, and the influence of interpersonal relationships [20]. The survey indicates that household income level, awareness of the hazards of influenza and the efficacy of vaccines, evaluation of vaccine safety, and health protection awareness are the primary factors affecting vaccination willingness, which also exert an impact on the actual vaccination rate to a certain extent [21]. Another study targeting medical staff in tertiary general hospitals revealed that despite a relatively high vaccination intention, such high intention did not automatically translate into high vaccination uptake. The conversion process was comprehensively influenced by occupational structural characteristics and external environmental factors, resulting in an obvious gap between intention and behavior [22]. This is mainly attributed to insufficient knowledge, safety concerns, and the financial burden caused by out-of-pocket vaccine costs. The current low vaccination rate is also closely associated with factors such as influenza vaccines not being included in the National Immunization Program and inadequate accessibility of vaccination services [15,22]. Despite the challenge of low vaccination rates, multiple provinces have adopted proactive measures and gradually explored various approaches to improve vaccination coverage, especially ensuring that key populations such as the elderly, children, and patients with chronic diseases can access vaccines in a timely manner [23]. For instance, since 2021, provinces and municipalities including Beijing, Shanghai and Guangdong have incorporated influenza vaccination into government livelihood projects. By means of government procurement of services and fixed subsidies, they have provided free or low-cost influenza vaccination for local residents aged 60 and above as well as primary and secondary school students. At present, Anhui Province adopts a self-funded and voluntary vaccination model, and only offers free influenza vaccination to people aged 65 and above and primary and secondary school students in some pilot areas. As local governments step up support and national immunization policies continue to improve, fairness and access to influenza vaccination will likely be further boosted. Such measures can help reduce the economic burden and make sure that target groups get vaccinated in a timely manner.

With the increasing number of influenza vaccine doses administered, post-marketing safety surveillance is critically important. The study focused on the surveillance data of AEFIs after InfV in Anhui Province, China, between 2016 and 2025. The results showed that the overall reporting rate of AEFIs induced by InfV was 34.40 per 100,000 doses, lower than the national rate of 70.1 per 100,000 doses in China. However, the reporting rate of rare adverse reactions was 1.16 per 100,000 doses, higher than the surveillance level of 0.4 per 100,000 doses in some Chinese provinces and cities [24]. This “dual-track” phenomenon indicates that while Anhui’s vaccination safety monitoring system is effective, it still faces challenges in the reporting and management of specific types of adverse reactions. The lower total incidence rate in Anhui may be attributed to the province’s highly sensitive AEFI surveillance network. Anhui has long implemented a combined strategy of passive surveillance and active case finding. The awareness of reporting among primary medical institutions for common reactions (such as fever and local redness/swelling) is strong, ensuring accurate denominators (vaccination doses) and a high volume of reports, thereby diluting the relative proportion of severe adverse events. In contrast, some regions in China may experience delays or under-reporting in their surveillance systems, leading to a statistically inflated proportion of severe events relative to the total. Due to limitations in data access, this study employed data derived from a meta-analysis of published literature for comparison; standardized cross-provincial data alignment remains unachievable at present. Joint multi-provincial monitoring and comparison undoubtedly represents a more rigorous scientific approach, offering new insights for future research. As a populous province, Anhui has a large registered population base according to the 2020 Population Census. During the study period, InfV vaccination in Anhui was primarily concentrated on priority groups, such as the elderly, children, and medical staff. These populations inherently carry a relatively higher burden of underlying diseases and exhibit more robust immune responses to vaccination, which may increase the absolute risk of individual severe adverse events. Furthermore, variations in the coverage rates of different InfV formulations, such as IIV3/4, LAIV3 may have influenced the distribution of AEFIs.

In this study, the reporting rate of common adverse reactions was 32.98 per 100,000 doses, mainly manifesting as fever, local redness, swelling, pain and induration. Most AEFI reports were concentrated within 24 h after vaccination, which is consistent with the findings of a study conducted in Beijing [25]. During the study period, influenza vaccination in this region was available to the general population, and the public health value of InfV has been widely recognized worldwide. Many regions in China have listed healthcare workers as priority groups for influenza vaccination. With the expansion of the target priority groups in the future, targeted AEFI surveillance is recommended to evaluate vaccine safety across different populations, thereby providing data support for comprehensive influenza prevention and control strategies. In addition, the first dose of vaccination was associated with the highest number of reported AEFI cases. This finding that AEFI incidence was higher after the first dose is consistent with our team’s previous research on HPV vaccines. The elevated reporting may be attributed to the relatively robust immune response triggered by initial exposure to vaccine antigens, whereas subsequent doses may induce greater immune tolerance, thereby leading to a lower incidence of adverse events. The reporting rate of rare adverse reactions to InfV was 1.16 per 100,000 doses. Among rare adverse reactions, allergic reactions constituted the predominant type, among which allergic rash was the most common, with a reporting rate of 0.98 per 100,000 doses. Individuals with a history of allergies should be closely monitored by healthcare professionals following vaccination to mitigate the risk of severe allergic reactions, including anaphylaxis. Individuals with a history of allergies, especially allergies to vaccine components, should undergo rigorous evaluation and make cautious decisions before influenza vaccination, and require close monitoring after inoculation. One case of Guillain–Barré Syndrome (GBS) was detected among neurological disorders, with a reporting rate of 0.01 per 100,000 doses. No other AEFIs such as meningitis were identified. The incidence of these neurological disorders is extremely low, and no definitive causal relationship between GBS and influenza vaccination was found in this study. In addition, cases including angioedema and febrile convulsions were reported in this study. Despite their low incidence, these AEFIs still demand focused attention, particularly when relevant symptoms occur post-vaccination. Most AEFIs in this study could be relieved with appropriate management or spontaneous recovery, further supporting the favorable safety profile of influenza vaccines. Although the proportion of severe AEFIs remains low, their potentially detrimental health impacts on individuals should not be overlooked. Accordingly, sustained surveillance, precise diagnosis, and prompt intervention of AEFIs are of great importance. Moving forward, the national AEFI surveillance system ought to be further refined to enhance the detection and reporting of severe adverse events, thereby ensuring timely recognition and effective clinical management. Moreover, in-depth investigations and comprehensive analyses of reported AEFI cases are warranted to elucidate underlying etiologies and relevant risk factors. Such efforts will support the development and implementation of targeted preventive strategies in routine immunization programs, ultimately minimizing the occurrence and burden of AEFIs.

Overall, the surveillance data on AEFIs associated with InfV in Anhui Province, China, during the period from 2016 to 2025 demonstrate that InfV is generally safe for widespread use in the target population. Nevertheless, the existing vaccination surveillance system still has room for continuous improvement to enhance its sensitivity, timeliness, and comprehensiveness in capturing potential adverse events. Meanwhile, to further improve influenza vaccination coverage and optimize influenza prevention and control strategies, it is crucial to sustain efforts in promoting influenza vaccination and carry out targeted public health education campaigns. Specifically, tailored health communication strategies should be developed for different age groups: for children, the focus should be placed on raising parents’ awareness of the necessity and safety of influenza vaccination, addressing their concerns about potential risks; for the elderly, efforts should be directed at educating them on the importance of timely vaccination, as well as its significant role in reducing the risk of severe influenza and related complications. These targeted measures are expected to improve public recognition and acceptance of influenza vaccines, thereby alleviating public vaccine hesitancy and increasing vaccination willingness. In addition, it is necessary to strengthen active surveillance of AEFIs, which can facilitate the timely identification of potential safety signals related to InfV and enable prompt intervention. Future research should prioritize in-depth investigations into AEFIs induced by different types of InfV, particularly those occurring after long-term and large-scale vaccination, so as to ensure the sustained safety and efficacy of InfV in practical application. Furthermore, it is recommended that influenza vaccines be gradually incorporated into provincial-level, and even national-level, immunization programs. To effectively increase vaccination rates, supportive policies such as government-led procurement and financial subsidies can be implemented to reduce the economic burden of vaccination for the public, enabling more individuals to access and benefit from influenza vaccination. Ultimately, these comprehensive measures will contribute to achieving better influenza prevention and control outcomes and safeguarding long-term public health security.

## 5. Conclusions

The findings of this study reveal that the overall reported incidence of suspected AEFIs subsequent to InfV administration in Anhui Province was relatively low. The majority of these adverse reactions were classified as common adverse events, including fever, local redness, swelling, and induration. In contrast, anaphylactic reactions accounted for the primary type of severe adverse events, though their incidence remained low. Notably, most AEFIs occurred following the first vaccine dose, and the vast majority of these events developed within 24 h after vaccination—findings that the reported incidence rate of severe adverse events is lower than the national surveillance level (1.64 per 100,000 cases) [26].

Nevertheless, the passive surveillance system employed in this research has certain inherent limitations. These limitations include underreporting of AEFIs and regional disparities in the capacity for investigation and diagnosis, which may result in an underestimation of the actual incidence of adverse events associated with InfV. Moving forward, the surveillance system can be further refined by implementing targeted active surveillance measures, enhancing the sensitivity and accuracy of surveillance efforts, and minimizing the occurrence of underreporting. Additionally, it is recommended to strengthen the capacity for AEFI investigation and diagnosis, improve standardized diagnostic criteria, and conduct long-term safety follow-up among vaccine recipients. These measures will collectively provide robust scientific evidence to support the optimization of influenza vaccination strategies.

As a vaccine not included in the national immunization program, InfV is not part of the mandatory national immunization schedule. Even so, expanding vaccination coverage—on the premise of ensuring vaccination safety through public health education and active public engagement—will help accelerate the establishment of population-level immune barriers. Ultimately, this will contribute to achieving the public health objective of reducing the overall transmission risk of influenza.

## Figures and Tables

**Table 1:** The incidence rate of AEFIs reported per 100,000 doses of InfV in Anhui Province, China, from 2016 to 2025.

Type	Common Adverse Reaction		Rare Adverse Reaction		Coincidental Event		Psychogenic Reaction		Total	
	NO.	Incidence *	NO.	Incidence	NO.	Incidence	NO.	Incidence	NO.	Incidence
IIV4(Split)	2562	36.19	47	0.66	13	0.18	5	0.07	2627	37.11
IIV3(Split)	1192	29.28	83	2.04	9	0.22	0	0.00	1284	31.54
LAIV3	62	15.57	6	1.51	1	0.25	0	0.00	69	17.33
IIV(Subunit)	44	28.09	0	0.00	2	1.28	0	0.00	46	29.37
Total	3860	32.98	136	1.16	25	0.21	5	0.04	4026	34.40

* Per 100,000 doses.

**Table 2:** Distribution characteristics of InfV-associated AEFIs in Anhui Province, China, 2016–2025.

Characteristics	IIV4(Split)		IIV3(Split)		LAIV3		IIV(Subunit)		Total	
	NO.	Proportion (%)	NO.	Proportion (%)	NO.	Proportion (%)	NO.	Proportion (%)	NO.	Proportion (%)
Gender										
male	1254	47.74	688	53.58	39	56.52	22	47.82	2003	49.75
female	1373	52.26	596	46.42	30	43.48	24	52.17	2023	50.25
Age										
<3	539	20.52	985	76.71	0	0.00	6	8.70	1530	38.00
3–17	1337	50.89	232	18.07	69	100.00	25	36.23	1663	41.31
18–59	442	16.83	49	3.82	0	0.00	12	17.39	503	12.49
≥60	309	11.76	18	1.40	0	0.00	3	4.35	330	8.20
Dose										
1	1867	71.07	977	76.09	60	86.96	35	76.09	2939	73
2	321	12.22	248	19.31	5	7.25	3	6.52	577	14.33
3	196	7.46	21	1.64	2	2.9	3	6.52	222	5.51
Time Interval										
≤1	2485	94.59	1219	94.94	60	86.96	40	86.96	3804	94.49
2–7	132	5.02	57	4.44	8	11.59	6	13.04	203	5.04
8–14	8	0.30	1	0.08	0	0.00	0	0.00	9	0.22
≥15	2	0.07	6	0.47	1	1.45	0	0.00	9	0.22

**Table 3:** Reporting Incidence Rates of Common Adverse Reactions Following Administration of Different InfV in Anhui Province, China, 2016–2025.

Clinical Diagnosis	IIV4(Split)		IIV3(Split)		LAIV3		IIV(Subunit)		Total	
	NO.	Incidence *	NO.	Incidence	NO.	Incidence	NO.	Incidence	NO.	Incidence
Common Adverse Reaction										
Fever (Axillary, °C)	1682	23.76	974	23.93	50	12.56	32	20.43	2738	23.39
≥38.6	619	8.74	474	11.64	21	5.27	13	8.30	1127	9.62
37.6–38.5	655	9.25	391	9.61	22	5.52	10	6.39	1078	9.21
37.1–37.5	408	5.76	109	2.68	7	1.76	9	5.75	533	4.55
Swelling (Diameter, cm)	1059	14.96	298	7.32	2	0.50	17	10.85	1376	11.77
>5.0	184	2.60	40	0.98	0	0.00	5	3.19	229	1.96
2.6–5.0	465	6.57	131	3.22	1	0.25	7	4.47	604	5.16
≤2.5	410	5.79	127	3.12	1	0.25	5	3.19	543	4.64
Induration (Diameter, cm)	771	10.89	197	4.84	1	0.25	13	8.30	982	8.39
>5.0	115	1.62	21	0.52	0	0.00	4	2.55	140	1.20
2.6–5.0	352	4.97	78	1.92	1	0.25	7	4.47	438	3.74
≤2.5	304	4.29	98	2.41	0	0.00	2	1.28	404	3.45
Others	10	0.14	1	0.01	1	0.01	1	0.01	13	0.11

* Per 100,000 doses.

**Table 4:** Reported incidence of rare adverse reactions following vaccination with different InfV in Anhui Province, China, from 2016 to 2025.

Clinical Diagnosis	IIV4(Split)		IIV3(Split)		LAIV3		IIV(Subunit)		Total	
	NO.	Incidence *	NO.	Incidence	NO.	Incidence	NO.	Incidence	NO.	Incidence
Allergic rash	39	0.55	71	1.74	5	1.25	0	0.00	115	0.98
Angioedema	2	0.03	4	0.10	1	0.25	0	0.00	7	0.05
Anaphylactic shock	3	0.03	1	0.02	0	0.00	0	0.00	4	0.03
Febrile convulsions	0	0.00	3	0.07	0	0.00	0	0.00	3	0.03
Anaphylactoid purpura	1	0.01	1	0.02	0	0.00	0	0.00	2	0.02
Thrombocytopenic purpura	1	0.01	1	0.02	0	0.00	0	0.00	2	0.02
Epilepsy	0	0.00	1	0.02	0	0.00	0	0.00	1	0.01
Guillain–Barré syndrome (GBS)	1	0.01	0	0.00	0	0.00	0	0.00	1	0.01
Aseptic abscess	0	0.00	1	0.02	0	0.00	0	0.00	1	0.01
Total	47	0.66	83	2.04	6	1.51	0	0.00	136	1.16

* Per 100,000 doses.

## Data Availability

Data used for this study are not publicly available due to confidentiality restrictions.
